# Supplementary material for: Evaluation of Class IIa Histone Deacetylases Expression and In Vivo Epigenetic Imaging in a Transgenic Mouse Model of Alzheimer’s Disease
Source: Int J Mol Sci. 2021 Aug 11;22(16):8633. doi: 10.3390/ijms22168633 (PMC8395513; doi:10.3390/ijms22168633)
Supplement: Supplementary file 1 [file ijms-22-08633-s001.zip › ijms-1288722-SI.pdf]

Evaluation of Histone Deacetylases Class IIa Expression and In Vivo Epigenetic Imaging in a Transgenic Mouse Model for Alzheimer's disease

Yi-An Chen [1,2,3], Cheng-Hsiu Lu [2,4], Chien-Chih Ke [5,6,7,*], Sain-Jhih Chiu [2], Chi-Wei Chang [8], Bang-Hung Yang [8,9], Juri G. Gelovani [10] and Ren-Shyan Liu [1,2,3,4,9,*]

Affiliations:

[1]Institute of Clinical Medicine, National Yang Ming Chiao Tung University, Taipei 112, Taiwan

[2]Molecular and Genetic Imaging Core/Taiwan Mouse Clinic, National Comprehensive Mouse Phenotyping and Drug Testing Center, Taipei 112, Taiwan

[3]Department of Nuclear Medicine, Cheng Hsin Hospital, Taipei 112, Taiwan

[4]Industrial Ph.D Program of Biomedical Science and Engineering, National Yang Ming Chiao Tung University, Taipei 112, Taiwan

[5]Department of Medical Imaging and Radiological Sciences, Kaohsiung Medical University, Kaohsiung 807, Taiwan

[6]Department of Medical Research, Kaohsiung Medical University Hospital, Kaohsiung 807, Taiwan

[7]Drug Development and Value Creation Research Center, Kaohsiung Medical University, Kaohsiung 807, Taiwan

[8]National PET and Cyclotron Center (NPCC), Department of Nuclear Medicine, Taipei Veterans General Hospital, Taipei 112, Taiwan

[9]Department of Biomedical Imaging and Radiological Sciences, National Yang Ming Chiao Tung University, Taipei 112, Taiwan

[10]Office of the Provost, United Arab Emirates University, Al Ain, United Arab Emirates

*Correspondence: rsliu@vghtpe.gov.tw (R.-S.L.); ccke@kmu.edu.tw (C.-C.K.)

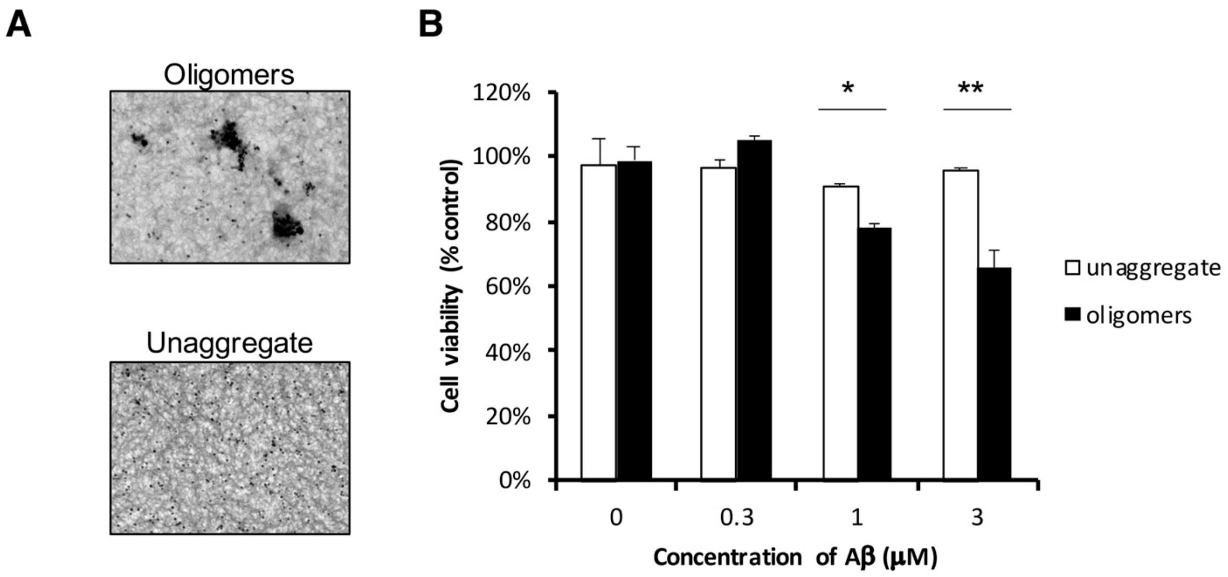

Supplementary figure 1. Neurotoxicity of Aβ oligomers to SH-SY5Y human neuroblastoma cells. (A) TEM analysis of Aβ oligomers and unaggregated $A\beta_{42}$. Accelerating voltage 100 kV and magnification 25000 times were used in this work. (B) Unaggregated and oligomers of $A\beta_{42}$ were incubated with SH-SY5Y cells for 48 hours. The 0.3, 1, 3 μM of $A\beta_{42}$ was diluted directly into cell culture media. The CCK8 assay was used as an indicator of cell viability. $A\beta_{42}$ oligomers reduced neuronal viability significantly more than unaggregated Aβ. $^{*}p < 0.05$ and $^{**}p < 0.01$ by Student's *t* test. Abbreviation: TEM, transmission electron microscopy.

A

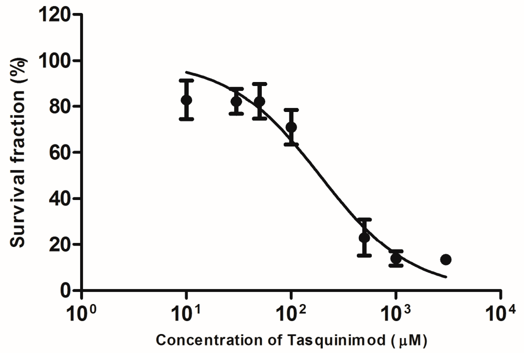

B

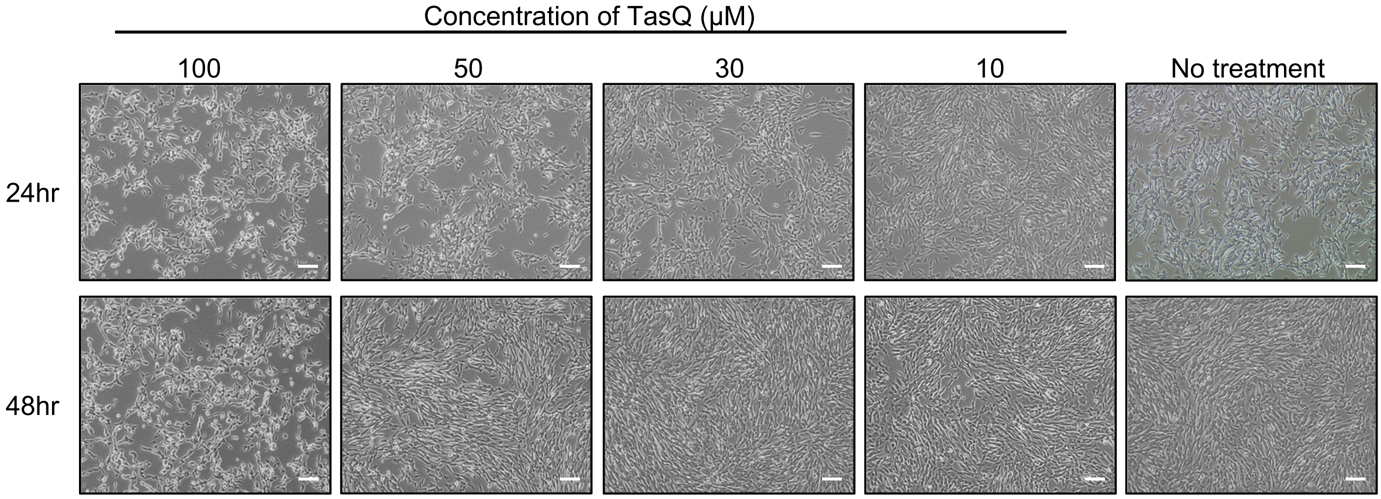

Supplementary figure 2. Toxicity of HDAC4 selective inhibitor-Tasquinimod (TasQ). (A) Cell viability was assessed by CCK-8 assays. All data were normalized to cells without treatment. CCK-8 assays showed that the IC_{50} of TasQ on SH-SY5Y-FAD cell line was 243 μM, N = 3. Data are presented as mean ± SD. (B) The cell morphology did not significantly change at lower concentration (10-50 μM) until the dose was increased to 100 μM (high concentration). Scale bar, 100 μm.

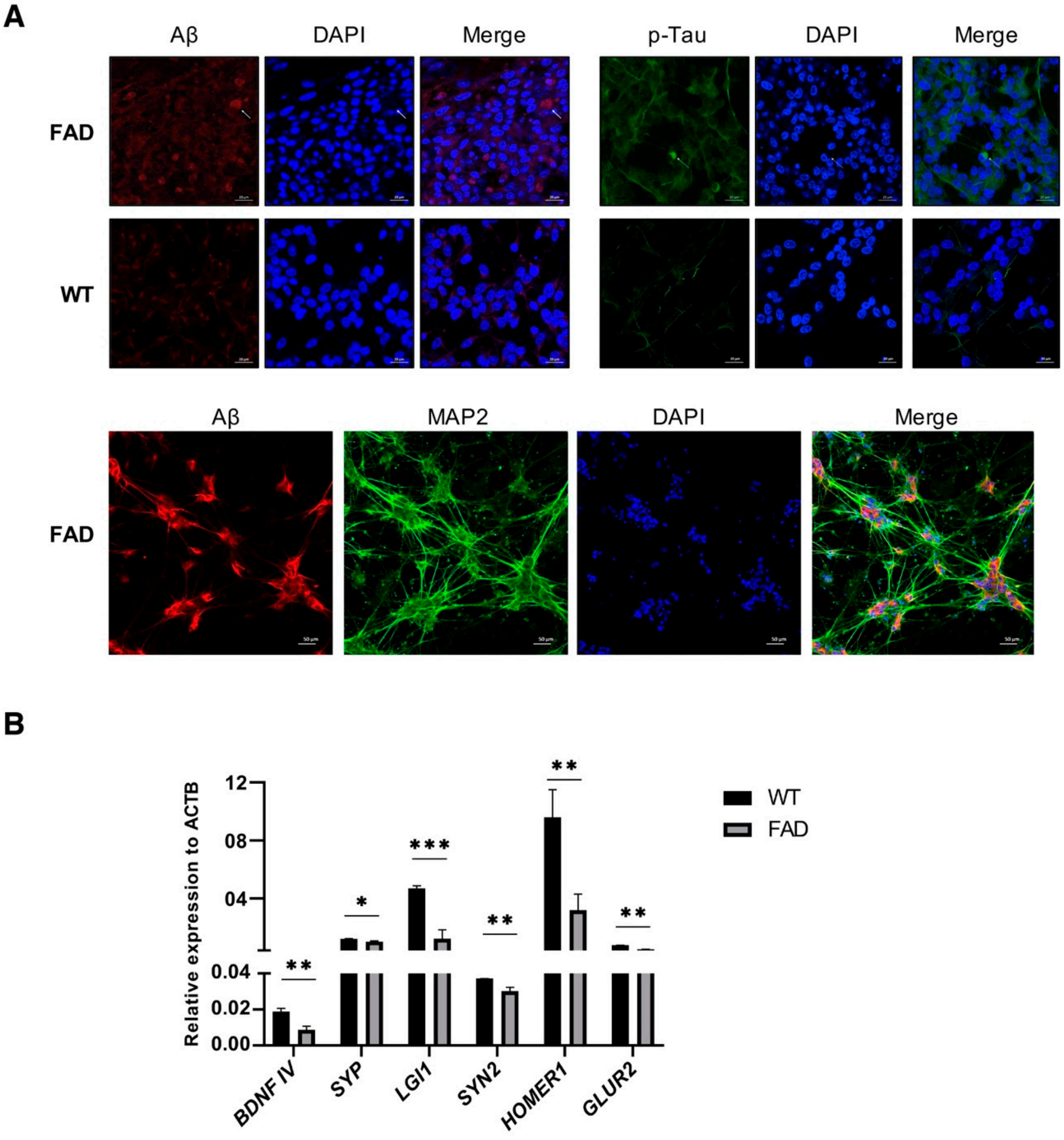

Supplementary figure 3. Establishment of FAD human neural cell culture model. (A) IF staining against β-amyloid and p-tau (S396 and S404) after differentiation. Blue, DAPI; scale bar, 20 μm. Detection of mature neuron marker, MAP2 and amyloid-β. Scale bar, 50 μm. (B) Quantitative RT-PCR results of neuron and plasticity-related genes in the FAD human neural cell culture model. Data are expressed as mean ± SEM (*$p < 0.05$, **$p < 0.01$, ***$p < 0.005$ by Student's t test). N.D, no detected; N.S, nonsignificant.

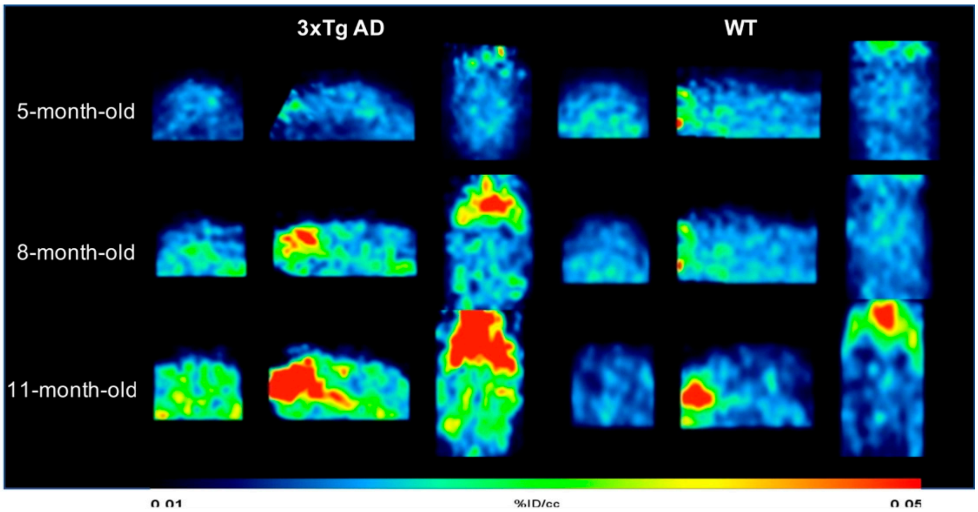

Supplementary figure 4. Aβ deposition using [^{11}C]PiB-PET imaging in 3xTg AD mice. Representative [^{11}C]PiB PET imaging of 3xTg AD mice and WT mice at 5, 8 and 11 months of age respectively. Coronal and sagittal slices were projected on built-in T1 MRI mouse template of Pmod software.

A

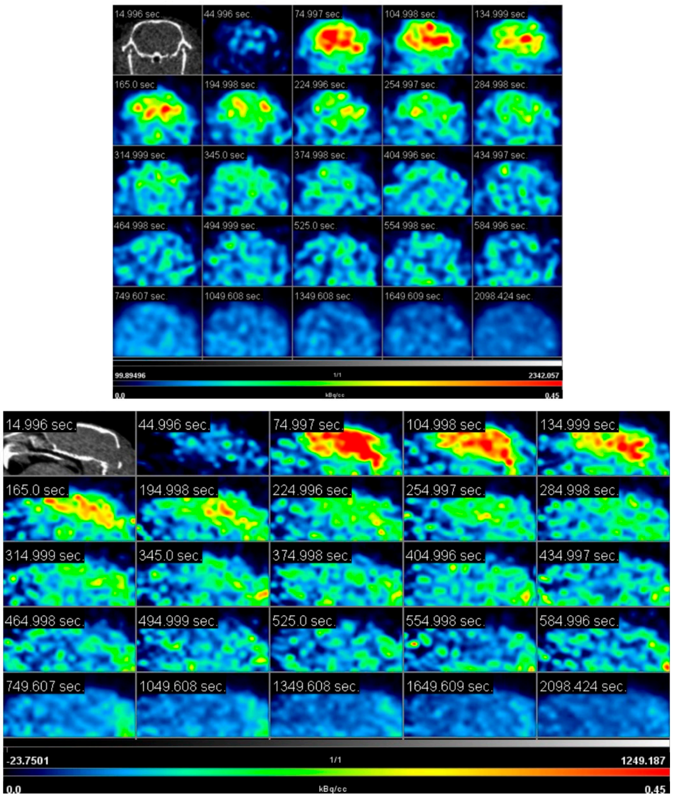

B

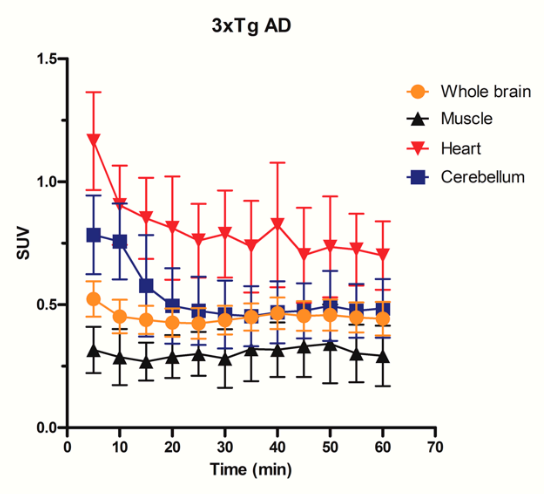

Supplementary figure 5. Dynamic PET imaging of [^{18}F]TFAHA in 3xTg AD mice. (A) Dynamic PET imaging following intravenous administration of [^{18}F]TFAHA. Coronal and sagittal slices were co-registered to the CT images by Pmod software (scale by %ID/c.c). (B) Time dependent distribution of [^{18}F]TFAHA.

A

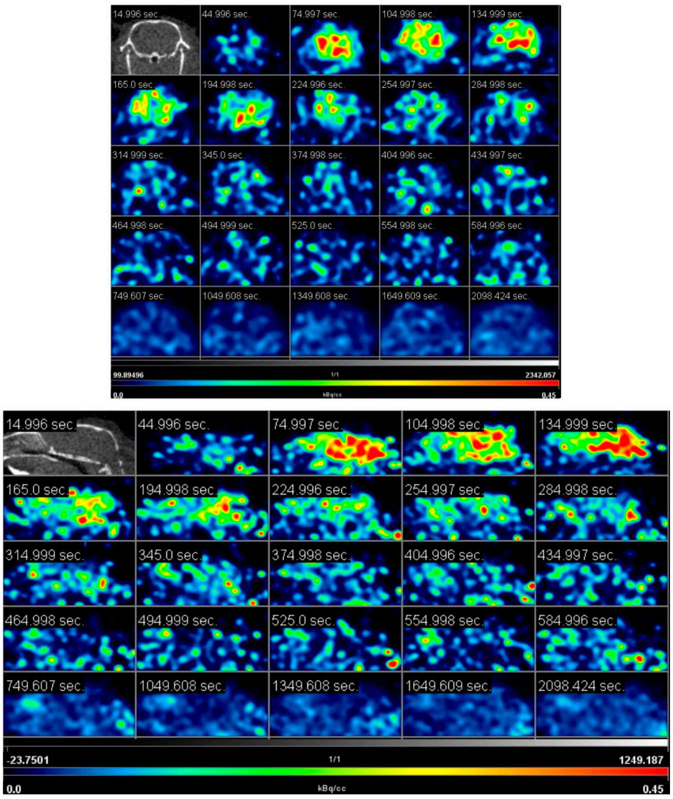

B

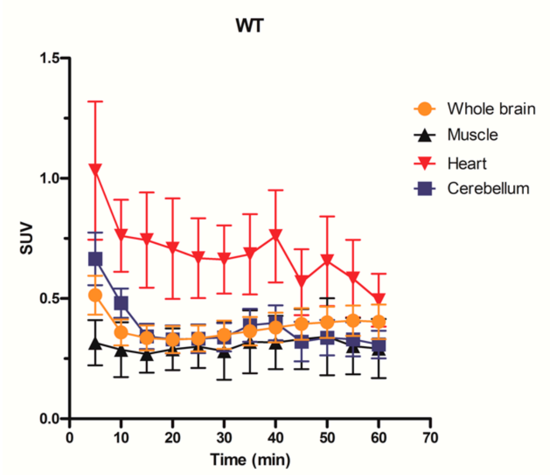

Supplementary figure 6. Dynamic PET imaging of [^{18}F]TFAHA in WT mice. (A) Dynamic PET imaging following intravenous administration of [^{18}F]TFAHA. Coronal and sagittal slices were co-registered to the CT images by Pmod software (scale by %ID/c.c). (B) Time dependent distribution of [^{18}F]TFAHA.

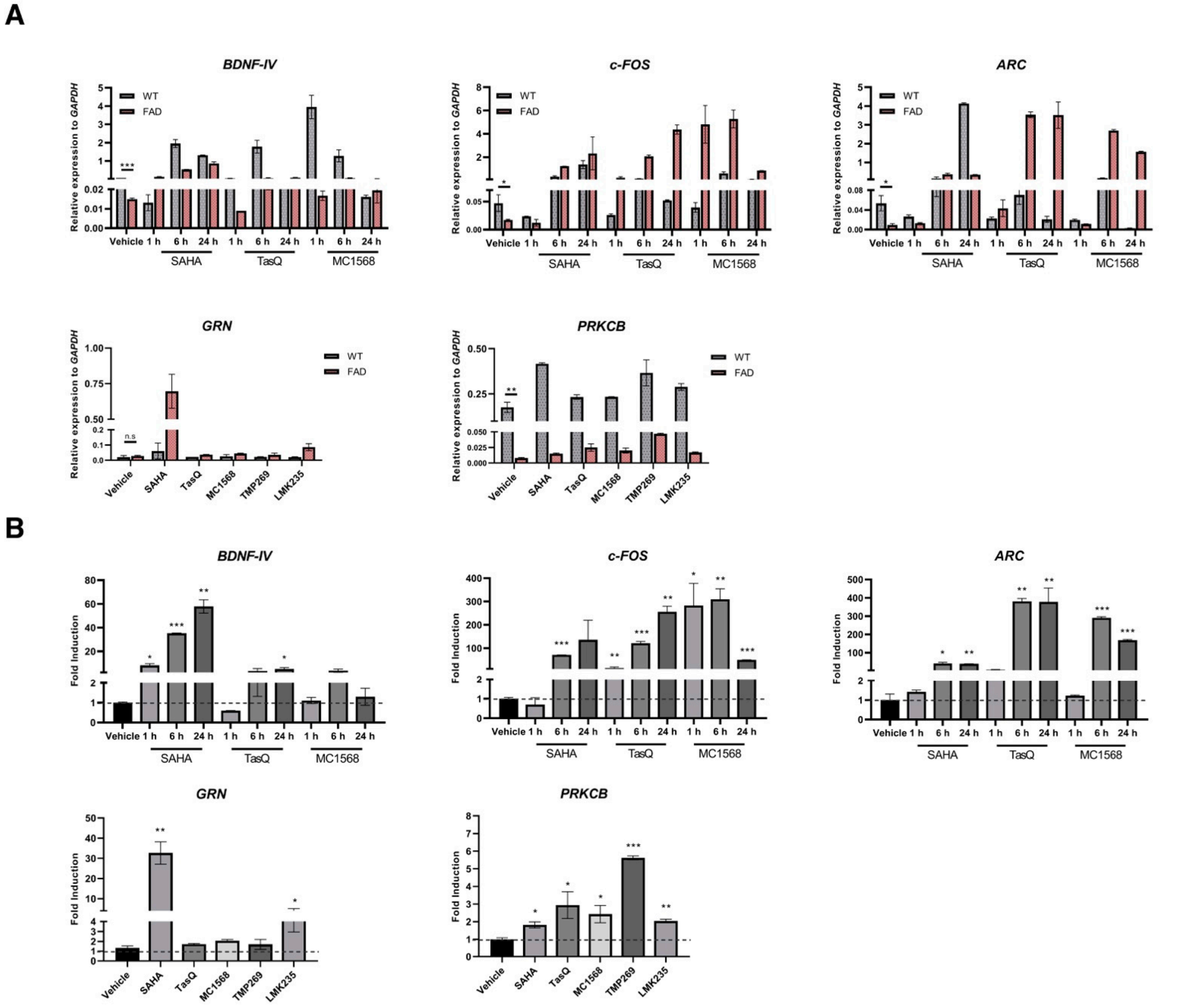

Supplementary figure 7. Comparison of the effects of class-selective HDAC inhibitors on memory/synaptic genes expression in FAD differentiated cells. Differentiated FAD cells and WT cells were treated with HDAC inhibitors (10 μM) or vehicle (DMSO) and analyzed mRNA levels by qRT-PCR at indicated time points after treatment. The analysis on GRN and PRKCB gene after the treatment of HDAC inhibitors for 24h. Gene expression levels were normalized against GAPDH levels (housekeeping gene) in each sample. Values are means ± SD of triplicate well. Statistics were analyzed by unpaired Student's *t*-test. $^*p < 0.05$, $^{**}p < 0.01$, $^{***}p < 0.001$, ns, nonsignificant. (A) Gene expression levels in differentiated FAD cells and WT cells. (B) The results were expressed as fold induction relative to vehicle controls in differentiated FAD cells. $^*p < 0.05$, $^{**}p < 0.01$, $^{***}p < 0.001$, significant increase relative to vehicle controls (*t*-test).

	3xTg AD		WT	
**Brain regions/months of age**	8	11	8	11
Striatum	0.471±0.026	0.665±0.067	0.420±0.08	0.386±0.051
Cortex	0.416±0.015	0.557±0.041	0.373±0.058	0.41±0.044
Hippocampus	0.435±0.034	0.582±0.046	0.359±0.063	0.354±0.042
Basal forebrain	0.469±0.040	0.680±0.080	0.420±0.093	0.408±0.059
Thalamus	0.463±0.026	0.629±0.060	0.379±0.071	0.387±0.037
Hypothalamus	0.446±0.041	0.651±0.041	0.388±0.064	0.400±0.075
Amygdala	0.447±0.047	0.621±0.041	0.409±0.061	0.380±0.042
Olfactory bulb	0.419±0.022	0.662±0.061	0.396±0.061	0.407±0.097
Cerebellum	0.396±0.018	0.523±0.053	0.342±0.037	0.356±0.04
Whole brain	0.433±0.028	0.604±0.015	0.377±0.013	0.371±0.013

Supplementary Table 1. Region-of-interest (ROI) analysis of PET images. Results were expressed as the percentage injected dose per cubic centimeter of tissue (%ID/c.c). Each value represented mean ± SD.

Inhibitor	Gene name	Treatment hour (s)	Gene expression (Normalized against GAPDH)		*P* value (WT vs. FAD)
			**WT**	**FAD**	
SAHA	BDNF IV	1	0.0131±0.0041	0.1195±0.0226	0.0112
		6	1.9510±0.2176	0.5301±0.0033	0.0058
		24	1.3228±0.0097	0.8698±0.0850	0.0087
	C-FOS	1	0.0237±0.0001	0.0120±0.0059	0.0527
		6	0.2982±0.0858	1.2134±0.0061	0.0022
		24	1.3595±0.3512	2.3221±1.4195	0.2251
	ARC	1	0.0262±0.0034	0.0132±0.0009	0.0171
		6	0.1454±0.0784	0.3671±0.0575	0.0421
		24	4.1383±0.0310	0.3474±0.0061	<0.0001
Tasquinimod (TasQ)	BDNF IV	1	0.0392±0.0073	0.0090±0.0001	0.0141
		6	1.7806±0.3507	0.0491±0.0293	0.0100
		24	0.0272±0.0001	0.0750±0.0181	0.0323
	C-FOS	1	0.0260±0.0018	0.2317±0.0773	0.0320
		6	0.1420±0.0080	2.0695±0.1232	0.0010
		24	0.0521±0.0007	4.3711±0.3979	0.0021
	ARC	1	0.0225±0.0029	0.0434±0.0171	0.1154
		6	0.0703±0.0191	3.5372±0.1511	0.0005
		24	0.0207±0.0061	3.5161±0.7016	0.0098
MC1568	BDNF IV	1	3.9480±0.6416	0.0167±0.0024	0.0065
		6	1.2789±0.3282	0.0527±0.0212	0.0171
		24	0.0161±0.0009	0.0195±0.0065	0.2708
	C-FOS	1	0.0395±0.0087	4.8183±1.6190	0.0264
		6	0.5978±0.1276	5.2781±0.7672	0.0068
		24	0.0890±0.0043	0.8303±0.0104	0.0001
	ARC	1	0.0196±0.0016	0.0114±0.0003	0.0097
		6	0.1651±0.0078	2.6947±0.0594	0.0001
		24	0.0028±0.0006	1.5627±0.0373	0.0001
Vehicle	BDNF IV	24	0.0656±0.0007	0.0150±0.0005	<0.0001
	C-FOS	24	0.0469±0.0150	0.0165±0.002	0.0416
	ARC	24	0.0535±0.0155	0.0093±0.0028	0.0291

Gene name	Inhibitor	Gene expression (Normalized against GAPDH)		*P* value (WT vs. FAD)
		**WT**	**FAD**	
GRN	Vehicle	0.0212±0.0008	0.0285±0.0042	0.0684
	SAHA	0.0612±0.0523	0.6965±0.1185	0.0101
	Tasquinimod	0.0219±0.0000	0.0367±0.0016	0.0028
	MC1568	0.0264±0.0105	0.0443±0.0028	0.0727
	TMP260	0.0221±0.0017	0.0361±0.0110	0.1086
	LMK235	0.0213±0.0023	0.0859±0.0229	0.0291
PRKCB	Vehicle	0.1761±0.0280	0.0084±0.0008	0.0063
	SAHA	0.4157±0.0058	0.0153±0.0014	<0.0001
	Tasquinimod	0.2334±0.0124	0.0248±0.0064	0.0007
	MC1568	0.2346±0.0007	0.0205±0.0041	<0.0001
	TMP260	0.3660±0.0715	0.0473±0.0009	0.0095
	LMK235	0.2891±0.0183	0.0172±0.0009	0.0010

Inhibitor	Gene name	Treatment hour(s)	Fold Induction (Relative to vehicle control)	*P* value
SAHA	BDNF IV	1	7.95±1.50	0.0113
		6	35.28±0.22	<0.0001
		24	57.88±5.65	0.0025
	C-FOS	1	0.70±0.35	0.1755
		6	71.16±0.36	<0.0001
		24	136.18±83.25	0.0742
	ARC	1	1.42±0.09	0.1028
		6	39.49±6.19	0.0064
		24	37.37±0.65	<0.0001
Tasquinimod (TasQ)	BDNF IV	1	0.60±0.002	0.0113
		6	3.26±1.95	0.1210
		24	4.99±12.0	0.0125
	C-FOS	1	13.59±4.53	0.0018
		6	121.37±7.23	<0.0001
		24	256.34±23.33	0.0021
	ARC	1	4.66±1.84	0.0545
		6	380.48±16.25	0.0005
		24	378.20±75.46	0.0097
MC1568	BDNF IV	1	1.11±0.16	0.2178
		6	3.51±1.41	0.0642
		24	1.29±0.43	0.2173
	C-FOS	1	282.57±94.95	0.0262
		6	309.54±44.99	0.0052
		24	48.69±0.612	<0.0001
	ARC	1	1.23±0.03	0.2009
		6	289.85±6.39	<0.0001
		24	168.09±4.01	<0.0001

Gene name	Inhibitor	Fold (Relative to vehicle controls)	*P* value
GRN	SAHA	32.66±5.56	0.0077
	Tasquinimod	1.72±0.07	0.0598
	MC1568	2.08±0.13	0.2310
	TMP260	1.69±0.52	0.2276
	LMK235	4.03±1.08	0.0367
PRKCB	SAHA	1.82±0.17	0.0133
	Tasquinimod	2.95±0.76	0.0346
	MC1568	2.43±0.49	0.0276
	TMP260	5.62±0.11	0.0002
	LMK235	2.04±0.10	0.0043

Supplementary Table 2. The statistics for all the comparisons, Related to Figure S7.

Gene name	Encoded protein	Function	Specific primers for genes used in RT-qPCR
SYP	Synaptophysin	organizing synaptic vesicles with other membrane components	F: 5′-TGCGCTAGAGCATTCTGGG-3′<br>R: 5′-CTTAAAGCCTCGCCCCTTCT-3
GLUR2	Glutamate ionotropic receptor AMPA type subunit 2	Cell migration and calcium signaling	F: 5′-TTGAAAAACAAATGGTGGTACG-3′<br>R: 5′- CTGAGGGCACTGGTCTTTTC-3′
HOMER1	Homer protein homolog 1	Scaffold. KOs have synaptic and cognitive deficits	F: 5′- TCCAAATTGACCCAAACACA-3′<br>R: 5′- TTGCCTTTGAGCCATCTAAA-3′
LGI1	leucine-rich glioma inactivated 1	AMPA receptor trafficking	F: 5′-CAAAGGCCTGGATTCTTTAAC-3′<br>R: 5′-CAACAGTTGCATTGGTGTGG-3′
SYN2	Synapsin II	Key modulator in neurotransmitter release	F: 5′-CCTTGGAGATTATGATATCAAGGT-3′<br>R: 5′-GCCACCAGGTTGAGCTCTGA-3′
GRN	Granulin	The protein has been implicated in cell proliferation and protein homeostasis. One of the autosomal dominant forms of FTD caused by GRN gene mutations.	F: 5′-CTCTCCAAGGAGAACGCTACCA-3′<br>R: 5′-GACTGTAGACGGCAGCAGGTAT-3′
BDNF	Brain-derived neurotrophic factor	Essential for neuronal growth and differentiation of new neurons and synapses	F: 5′-CTCCGCCATGCAATTTCCAC-3′<br>R: 5′-GCCTTCATGCAACCGAAGTA-3′
C-FOS	Proto-oncogene	Immediate early genes that are associated with synaptic plasticity and memory formation plasticity	F: 5′-GCCTCTCTTACTACCACTCACC-3′<br>R: 5′-AGATGGCAGTGACCGTGGGAAT-3′
ARC	Activity regulated cytoskeleton associated protein	Immediate early genes that are associated with synaptic plasticity and memory formation	F: 5′-CTGAGCCACCTAGAGGAGTACT-3′<br>R: 5′-AACTCCACCCAGTTCTTCACGG-3′

PRKCB	Protein kinase C	Multiple forms of synaptic plasticity	F: 5′- TACTCCAGCCCCACGTTTTG-3′<br>R: 5′-TCACTTCCTTCTGGTGGCAC-3′
GAPDH	Glyceraldehyde 3-phosphate dehydrogenase	Housekeeping gene	F: 5′-CAACAGCTTGCTGCGTACC-3′<br>R: 5′-CTAACACACTCCAGCTCAGTGAC-3′
ACTB	Cytoskeletal actin	Housekeeping gene	F: 5′-GCCGGGACCTGACTGACTAC-3′<br>R: 5′-GGAACCGCTCATTGCCAAT-3′

Supplementary Table 3. Genes essential for synaptic function and memory formation, Related to Figure 3 and Figure S7.

Materials and methods

Establishment of FAD human neural cell culture model-Cont

Generation of stable lines by lentivirus infection: The 293T cells were transfected with the pLKO-AS3-APP_{695} (S/L)-EGFP, pCMV-ΔR8.91 and pMD.G to produce lentivirus. Thereafter, 1ml viral solution was added to 85% confluent SH-SY5Y cells in 6-well plate, incubated overnight, and replaced growth media with 1μg/ml puromycin. The expression of the infected genes was confirmed by GFP expression by fluorescence microscopy.

IF staining for AD markers: Briefly, after washing with PBS twice and fixing with 4% formaldehyde, cell culture slides were incubated with the primary antibodies overnight at 4°C, and then incubated with Alexa 594 or Cy5-conjugated secondary antibodies (Abcam, Cambridge, UK) and DAPI for 1 h in the dark room at room temperature prior to PBS washing for 3 times. All slides were mounted with ProLong Antifade Mounting Medium (catalog #P36970, Life Technologies, Carlsbad, CA, USA) and covered with cover slides. The primary antibodies and dilution rates were as follows: anti-Aβ antibody (1:400, 6E10, Covance Inc, Princeton, NJ, USA); anti-pTau antibody (Ser396 and Ser404, 1:200, Arigo, Taiwan); MAP2 (1:500, Millipore, Bedford, MA, USA).

Dynamic [^{18}F]TFAHA PET Imaging Procedures

Imaging was performed using a Triumph PET/SPECT/CT imaging scanner (Gamma Medica-Ideas, Northridge, CA, USA) for data acquisition and imaging process. Each mouse were placed in the positioning bed and anesthetized with 2% isoflurane in oxygen at 2L/ min followed by intravenously injection of 8.04±0.75 MBq/0.1 ml of [^{18}F]TFAHA. Dynamic PET images were obtained over 60 minutes, followed by overlapping frames (10 min each) acquired to obtain whole brain images of [^{18}F]TFAHA. Additional CT scan was performed for anatomic reference. Subsequently, regional retention and uptake of [^{18}F]TFAHA were processed and analyzed with PMOD 3.5 software package (Pmod Technologies, Zurich, Switzerland).

Cell Viability Assay

SH-SY5Y cells were seeded in a 96-well cell culture plate at the density of $1x10^4$ cells/well. Then, after incubation at 37 °C overnight, a series of concentrations of aggregated Ab_{42} and oligomers were added into the culture medium and co-incubated for 48 h. Finally, 10 μL of the CCK-8 reagent (Dojindo Molecular Technologies, Inc, Japan) was added into each well, and OD at 450 nm was measured using an absorbance microplate reader (Sunrise, Tecan, Zürich, Switzerland) after incubation for 2 h at 37 °C. The percentage of each concentration accounted for of the control was

presented as cell viability. To determine the neurotoxicity of Tasquinimod, a cell viability assay was performed as described above. FAD-differentiated were cultured for 7 days in a differentiation medium, followed by incubation with or without Tasquinimod at different concentrations for 48 h. IC_{50} values were calculated from a log([drug]) versus normalized response curve fit using GraphPad Prism version 5.0 (GraphPad Software).

Inhibitor treatments and reverse transcription and quantitative real-time polymerase chain reaction (PCR)

To compare the effects of five HDAC inhibitors of different selectivity on neuronal memory and synaptic plasticity-related genes, we treated differentiated FAD cells and WT cells with HDAC inhibitors respectively and analyzed mRNA levels by qRT-PCR at indicated time points after treatment. SAHA, MC1568, TMP269 and LMK235 were purchased from Cayman Chemical (Ann Arbor, Michigan, USA). HDAC inhibitors were dissolved in DMSO and added to neuronal cultures at a final concentration of 10 μM. The average threshold cycle (Ct) for each gene was normalized by that Ct of GAPDH.
